# Shaping Skeletal Growth by Modular Regulatory Elements in the *Bmp5* Gene

**DOI:** 10.1371/journal.pgen.1000308

**Published:** 2008-12-19

**Authors:** Catherine Guenther, Luiz Pantalena-Filho, David M. Kingsley

**Affiliations:** 1 Department of Developmental Biology, Stanford University School of Medicine, Stanford, California, United States of America; 2 Howard Hughes Medical Institute, Stanford University School of Medicine, Stanford, California, United States of America; Harvard Medical School, United States of America

## Abstract

Cartilage and bone are formed into a remarkable range of shapes and sizes that underlie many anatomical adaptations to different lifestyles in vertebrates. Although the morphological blueprints for individual cartilage and bony structures must somehow be encoded in the genome, we currently know little about the detailed genomic mechanisms that direct precise growth patterns for particular bones. We have carried out large-scale enhancer surveys to identify the regulatory architecture controlling developmental expression of the mouse *Bmp5* gene, which encodes a secreted signaling molecule required for normal morphology of specific skeletal features. Although *Bmp5* is expressed in many skeletal precursors, different enhancers control expression in individual bones. Remarkably, we show here that different enhancers also exist for highly restricted spatial subdomains along the surface of individual skeletal structures, including ribs and nasal cartilages. Transgenic, null, and regulatory mutations confirm that these anatomy-specific sequences are sufficient to trigger local changes in skeletal morphology and are required for establishing normal growth rates on separate bone surfaces. Our findings suggest that individual bones are composite structures whose detailed growth patterns are built from many smaller lineage and gene expression domains. Individual enhancers in BMP genes provide a genomic mechanism for controlling precise growth domains in particular cartilages and bones, making it possible to separately regulate skeletal anatomy at highly specific locations in the body.

## Author Summary

Every bone in the skeleton has a specific shape and size. These characteristic features must be under separate genetic control, because individual bones can undergo striking morphological changes in different species. Researchers have long postulated that the morphology of individual bones arises from the local activity of many separate growth domains around each bone's surface. Differential growth within such domains could modify size, curvature, and formation of specific processes. Here, we show that local growth domains around individual bones are controlled by independent regulatory sequences in bone morphogenetic protein (BMP) genes. We identify multiple regulatory sequences in the *Bmp5* gene that control expression in particular bones, rather than all bones. We show that some of these elements are remarkably specific for individual subdomains around the surface of individual bones. Finally, we show that local BMP signaling is necessary and sufficient to trigger highly localized growth patterns in ribs and nasal cartilages. These results suggest that the detailed pattern of growth of individual skeletal structures is encoded in part by multiple regulatory sequences in BMP genes. Gain and loss of anatomy-specific sequences in BMP genes may provide a flexible genomic mechanism for modifying local skeletal anatomy during vertebrate evolution.

## Introduction

The vertebrate skeleton is constructed of cartilage and bone tissues that are formed into highly specific shapes, sizes, and repeating arrays during normal development. Individual bones can show striking morphological specializations in different species, suggesting that separate genetic mechanisms must exist for regulating the growth of skeletal tissue at highly specific anatomical sites in the body [1],[2]. Despite the importance of skeletal structures for support, protection, eating, breathing, and movement, the detailed genetic mechanisms controlling the shape and growth of individual bones are still poorly understood.

Over fifty years ago, Bateman proposed that characteristic skeletal shapes are determined by varying patterns of differential growth and erosion that occur in stereotyped positions along the surfaces of each bone [3]. Localized growth at ends of a bone results in long straight structures. Uniform deposition around a bone produces uniform circumferential growth. Preferential deposition and erosion on opposite surfaces of a bone generates lateral displacement or curvature. Localized patches of deposition and erosion may also produce many of the specific processes, ridges, foramina, and articular surfaces that are characteristic of each bone in the body. Although highly localized patterns of deposition and erosion have long been proposed or visualized in the skeleton [4]–[6], little is known about how such stereotyped patterns may be encoded in the genome.

Previous studies demonstrate that secreted signaling molecules in the bone morphogenetic protein (BMP) family play a key role in both formation and repair of skeletal structures [7]. These molecules are expressed both in early skeletal precursors, and in the surface perichondrium and periosteum layers that surround growing cartilage and bone [8]–[13]. Pure recombinant BMPs can induce cartilage and bone formation when implanted at ectopic sites in animals [14],[15]. Conversely, mouse mutants missing members of the BMP family show defects in subsets of bone and cartilage elements. The classical mouse *short ear* locus encodes one of the mammalian BMP molecules (BMP5) [16]. Mutations at this locus reduce outer ear growth by disrupting the formation and activity of the surface perichondrium surrounding outer ear cartilage [17]. The same locus also controls the presence or absence of processes on specific vertebrae and the fibula, the morphology of the xiphoid process at the end of the sternum, the number of ribs along the vertebral column, and the total volume of the thoracic cavity [18]–[21].

A large number of spontaneous and induced *short ear* mutations suggest that the *Bmp5* locus is surrounded by large regulatory regions required for developmental expression patterns in bones and other tissues [22]–[24]. Here we carry out detailed enhancer surveys to test the regional specificity of regulatory sequences controlling *Bmp5* expression in skeletal tissues. Our studies suggest that stereotyped growth patterns along the surface of both ribs and nasal cartilages are controlled by highly specific “anatomy” elements in the *Bmp5* gene. These modular enhancers in BMP genes may provide a flexible basis for encoding the detailed growth and form of specific bones in the vertebrate skeleton.

## Results

### Partitioning of *Bmp5* Rib Perichondrium Expression by Multiple Regulatory Sequences

**Figure 1:**
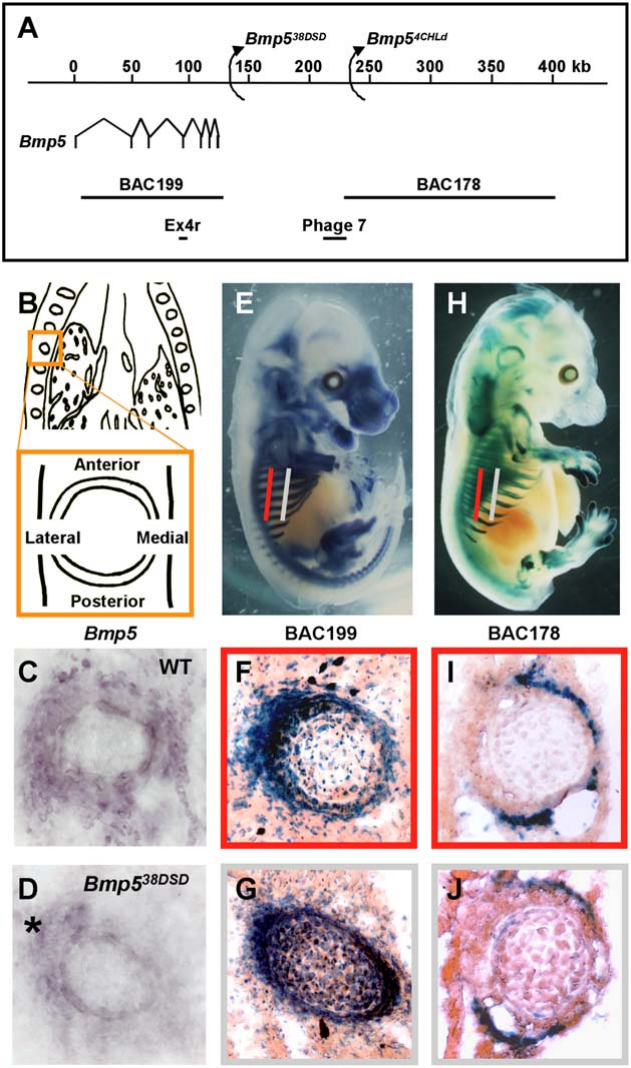
Multiple enhancers control *Bmp5* rib expression. A. Schematic of the *Bmp5* locus with positions of previously described regulatory alleles (*Bmp5^se38DSD^*, *Bmp5^se4CHLd^*), BACs, and the Ex4r and Phage 7 genomic clones used in transgenic analysis. B. Schematic of coronal sections through the torso and a rib (boxed) showing the orientation used in all rib cross-sections. C, D. *Bmp5 in situ*s on rib cryosections from E13.5 wild-type (C) and *Bmp5^se38DSD^* mutant (D) embryos. Note the reduced expression in the anterior, medial and posterior perichondrial domains of the *Bmp5^se38DSD^* rib, with continued expression in the lateral domain (asterisk). E. Lateral view of BAC199-*lacZ* transgenic embryo. Red and grey bars represent planes of dorsal (red) and ventral (grey) rib sections. F, G. Set of rib cryosections taken from dorsal (F) and ventral (G) planes from a BAC199 embryo. H. Lateral view of BAC178-*lacZ* transgenic embryo. I, J. Set of rib cryosections taken from dorsal (I) and ventral (J) planes from a BAC178 embryo. Overall *Bmp5* expression in rib perichondrium (C) is a mosaic pattern controlled by different regulatory regions for different spatial domains around the rib.

Previous regulatory scans of the *Bmp5* locus identified several large regions that could drive expression of a *lacZ* reporter gene in developing skeletal structures [23],[24]. Expression in developing ribs was observed when two different bacterial artificial clones (BACs) covering non-overlapping regions of the gene (Figure 1A, E, H) were coinjected with a minimal heat shock-*lacZ* expression construct [24]. BAC199 includes most of the exons and introns of the *Bmp5* gene. In contrast, BAC178 includes sequences from a large region three prime (3′) of all *Bmp5* coding exons. Chromosome rearrangements in this 3′ region have generated two regulatory alleles of the *Bmp5* locus (*Bmp5^se38DSD^* and *Bmp5^se4CHLd^*, Figure 1A). These alleles confirm that extensive 3′ sequences are required for normal expression and function of the endogenous *Bmp5* gene [23].

To compare the *lacZ* expression in ribs driven by different BACs spanning the *Bmp5* locus, we examined a series of coronal sections taken along the dorsoventral axis of the ribs of transgenic embryos beginning near the vertebral column and ending near the sternum. In dorsal sections from a BAC199-*lacZ* transgenic embryo, β-galactosidase activity was surprisingly restricted to a lateral domain within the rib perichondrium (Figure 1F). This pattern changed as sections progressed ventrally. In later sections, β-galactosidase activity was found in both the lateral and medial rib perichondrium (Figure 1G).

Interestingly, the pattern of *lacZ* expression controlled by the distal BAC clone, BAC178, was complementary to that seen with proximal BAC199. In dorsal sections from a BAC178-*lacZ* transgenic embryo, β-galactosidase activity was found in anterior, medial and posterior domains of the rib perichondrium (Figure 1I). More ventral rib sections showed loss of medial expression but retained β-galactosidase activity predominantly in anterior and posterior rib perichondrium (Figure 1J). Thus, BAC178-*lacZ* rib expression complements BAC199-*lacZ* rib expression as it changes along the dorsoventral axis (Figure 1F, I and G, J). Taken together, these results suggest that gene expression in different domains of the rib perichondrium is controlled by distinct regulatory elements in the *Bmp5* locus. Notably, the complementary rib regulatory regions are separated by over 100 kilobases (kb) (Figure 1A).

Analysis of endogenous *Bmp5* expression in wild-type and *Bmp5^se38DSD^* regulatory mutants confirms the existence of distinct control regions for different domains of the rib perichondrium. The *Bmp5^se38DSD^* regulatory mutation derives from a chromosomal rearrangement whose breakpoint lies near the *Bmp5* coding exons [22]. Therefore, this rearrangement is predicted to remove all distal rib control sequences (Figure 1A). *In situ* hybridization analysis of *Bmp5* transcripts in dorsal rib sections shows reduction of anterior, medial and posterior rib domain expression within *Bmp5^se38DSD^* ribs as compared to wild-type (Figure 1C, D). In contrast, strong *Bmp5* expression is still seen in the lateral rib perichondrium (asterisk in Figure 1D), as expected given the location of lateral control elements upstream of the *Bmp5^se38DSD^* breakpoint. Therefore, general *Bmp5* expression in rib perichondrium appears to be a composite of smaller, independently regulated expression domains.

**Figure 2 pgen-1000308-g002:**
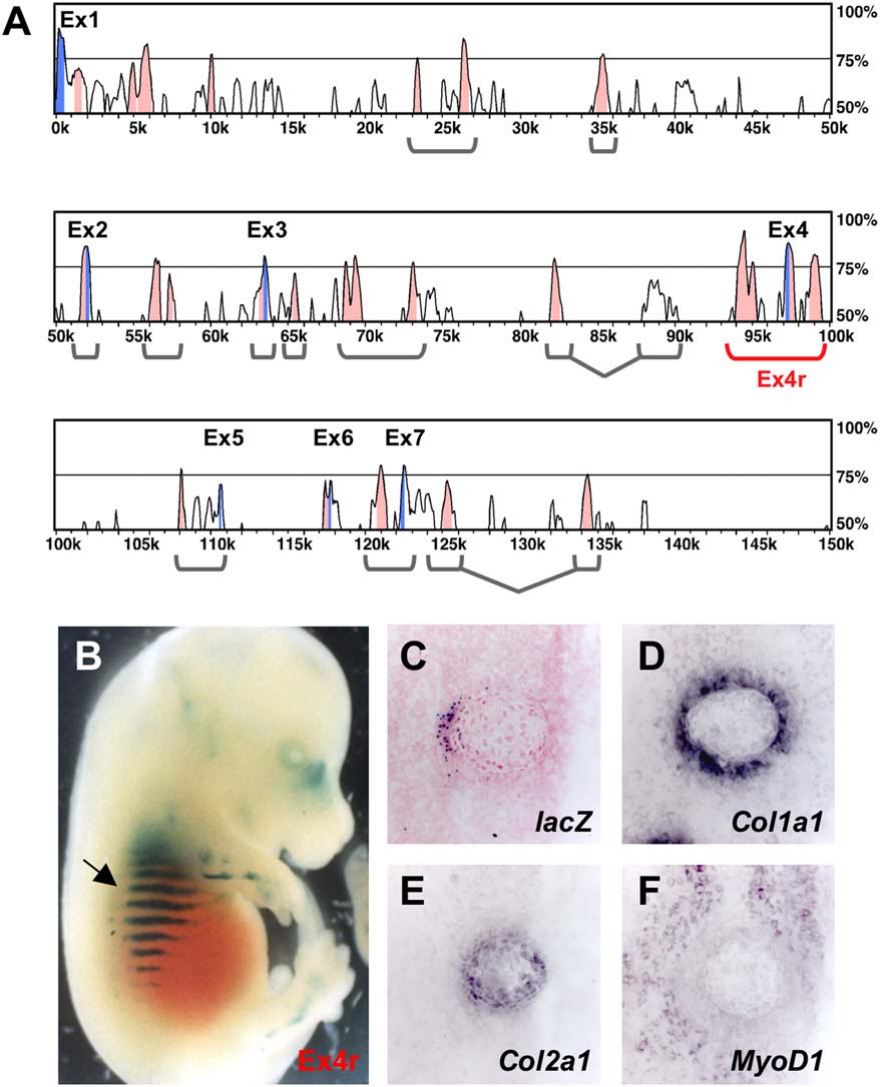
A 6.2 kb genomic subclone contains a lateral rib enhancer. A. Global sequence comparison of the mouse and human *Bmp5* loci by LAGAN/VISTA. Blue peaks denote exons with 0k representing the first nucleotide of *Bmp5* exon 1. ECRs of 70% identity over at least 300 bp are highlighted (pink). Percent identity (Y-axis) ranges from 50–100%. The gray and red brackets show ECR constructs from the BAC199 region (11.6 kb–128.9 kb) tested in the transgenic assay. B. The Ex4r genomic subclone (red bracket, A) gave consistent expression in nasal cartilages, limbs and ribs (arrow). C. Coronal section through the dorsal rib of an Ex4r-*lacZ* E14.5 transgenic embryo showing β-galactosidase activity. D–F. Near adjacent coronal sections from the same rib in panel C showing expression of *Col1a1* (perichondrium) (D), *Col2a1* (cartilage) (E), and *MyoD1* (skeletal muscle) (F). Comparison of panels C and D shows β-galactosidase activity controlled by the Ex4r subclone is predominantly restricted to the lateral perichondrium.

To further characterize and localize *Bmp5* regulatory sequences, we used sequence alignment programs PipMaker and LAGAN/VISTA to compare human and mouse *Bmp5* loci [25]–[27]. This approach revealed numerous evolutionarily conserved non-coding regions (ECRs) scattered across the *Bmp5* locus (Figure 2, Figure S1). We then cloned multiple small genomic fragments containing single or multiple ECRs upstream of a minimal heat shock-*lacZ* reporter cassette, injected them into fertilized mouse eggs, and scored expression patterns in transgenic embryos. The survey of putative enhancer regions extended across the entire 400 kb interval detailed in Figure 1A (see Figure 2A and Figure S1). A 6.2 kb clone from the BAC199 region including 4 ECRs surrounding *Bmp5* exon 4 (Ex4r) drove reproducible expression in nasal cartilages, distal limbs, and ribs (Figure 2B). As with the BAC transgenics, a series of coronal sections was taken along the ribs of Ex4r-*lacZ* transgenics. Dorsal rib sections again revealed expression in a restricted domain along the lateral surface of the developing rib (Figure 2C). To further characterize this peripheral surface domain we hybridized adjacent sections with molecular markers for perichondrium (type I collagen, *Col1a1*), chondrocytes (type II collagen, *Col2a1*, and type X collagen, *Col10a1*), and developing muscle (*MyoD1*) [28]–[30]. The *lacZ*-positive region corresponds to a particular sector of the surface perichondrium surrounding the ribs, which otherwise extends in a continuous circle around the developing rib cartilage (Figure 2C–F). Unlike the BAC199-*lacZ* transgene, the Ex4r sequence did not drive discrete localized expression in a medial perichondrium domain in more ventral rib sections (data not shown). These results demonstrate that anatomical control sequences for lateral perichondrium expression map within the exon 4 region, and that additional sequences are required for the medial rib expression seen with BAC199.

To further narrow the region required for lateral perichondrium expression, we tested a series of smaller genomic fragments and deletions of conserved ECRs from within the Ex4r subclone (Figure S2). This analysis demonstrated that the core sequences necessary for lateral perichondrium expression reside in a 1069 bp peak of conservation at the 3′ end of the Ex4r region, and that other sequences in the Ex4r construct are required for expression in limbs and nasal cartilages.

### Partitioning of *Bmp5* Nasal Cartilage Expression by Multiple Control Regions

**Figure 3 pgen-1000308-g003:**
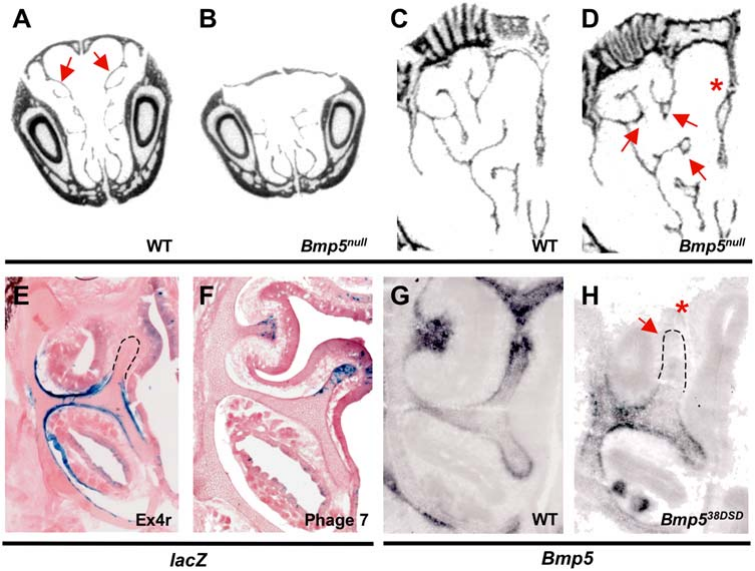
Coordinate regulation of *Bmp5* nasal cartilage expression by multiple enhancers. A–D. Two dimensional coronal microCT images from wild-type (A, C) and *Bmp5^null^* (B, D) mutants at 4 weeks. The anterior nasal turbinates (arrows, A) are missing from *Bmp5^null^* mutants (B). In more posterior regions of the nasal capsule, *Bmp5^null^* mutants (D) also exhibit malformations of the septum (asterisk) and tips of several turbinate branches (arrows) as compared to wild-type (C). E, F. Coronal cryosections through Ex4r-*lacZ* (E) and Phage7-*lacZ* (F) E16.5 transgenic nasal cartilage. β-galactosidase activity is limited to the turbinate necks and a small domain of cartilage between turbinate shelves in Ex4r-*lacZ* transgenics (E) while β-galactosidase activity appears only in the dorsal turbinate tips of Phage7-*lacZ* transgenics (F). G, H. *Bmp5 in situs* on coronal cryosections from E15.5 wild-type and *Bmp5^se38DSD^* mutants. *Bmp5* transcript is found throughout the turbinate shelf and in the cribriform plate, and is concentrated at the tips of growing turbinates in wild-type nasal cartilage. *Bmp5^se38DSD^* mutants (H) lack *Bmp5* expression in the dorsal turbinate tips (arrow) and the cribriform plate (asterisk). Outlines added to show position of turbinates lacking expression in E and H.

*Bmp5* is expressed in the perichondrium surrounding many other skeletal structures, including the nasal septum and the shelf-like turbinates that project into the nasal cavity [11]. In addition, new micro computerized tomography (MicroCT) analysis of wild-type and *Bmp5* mutant skulls shows that the *Bmp5* gene is also required for normal development of turbinates in the anterior nasal region (Figure 3A, B), and for normal branching patterns in more posterior nasal regions (Figure 3C, D). To determine whether *Bmp5* expression in nasal cartilages is also controlled by separable regulatory sequences, we examined the nasal region in both BAC199-*lacZ* and Ex4r-*lacZ* transgenic embryos. The larger BAC199 clone showed widespread β-galactosidase activity throughout the nasal cartilages, including multiple turbinates and the nasal septum (data not shown). In contrast, Ex4r-*lacZ* transgenics showed activity restricted to a small arc-like domain located on the inner surface of nasal cartilage between turbinate shelves in the anterior nasal cavity and along the neck of the developing turbinates (Figure 3E). Like the lateral rib expression, turbinate expression was seen predominantly in subregions of the surface perichondrium (Figure S3A, C). No expression was seen in the nasal septum, in posterior cartilages or at the tips of the shelf-like projections of the turbinates themselves. Testing fragments of the Ex4r clone demonstrated that sequences directing restricted nasal cartilage expression and restricted lateral rib perichondrium expression are distinct (Figure S2).

Examination of other regions of the *Bmp5* locus known to contain skeletal enhancers identified an additional non-overlapping sequence that also gives expression in nasal cartilages. A 17 kb clone (Phage 7 in Figure 1) previously reported to give thyroid cartilage expression [23] also showed strikingly specific nasal cavity expression. β-galactosidase activity was seen at the dorsal tips of the expanding turbinate shelves, colocalizing with *Col2a1* in proliferating chondrocytes, but was absent from the ventral tips, the turbinate necks, and the cartilages between turbinate shelves (Figure 3F, Figure S3B, F), a pattern partially complementary to that driven by the Ex4r construct.

The sequences included in Phage 7 are located approximately 100 kb 3′ of the chromosome breakpoint in the *Bmp5^se38DSD^* regulatory mutation (Figure 1A). Endogenous *Bmp5* expression is dramatically reduced in the dorsal tips of turbinate shelves in *Bmp5^se38DSD^* mice compared to wild-type (Figure 3G, H), as well as in the cribriform plate, the structural roof of the nasal cavity. These data confirm that 3′ regulatory sequences are required for *Bmp5* expression in the tips of turbinate shelves, but not in the surrounding neck and inter-turbinate perichondrium. *Bmp5^se38DSD^* mutant mice also show defects in the cribriform plate and branching alterations in nasal turbinates (data not shown). Thus, in both ribs and nasal cartilage, an apparently continuous layer of perichondrium consists of distinct expression domains controlled by separate regulatory elements in the *Bmp5* gene.

### Somite Compartments as Precursors of Rib Expression Domains

**Figure 4:**
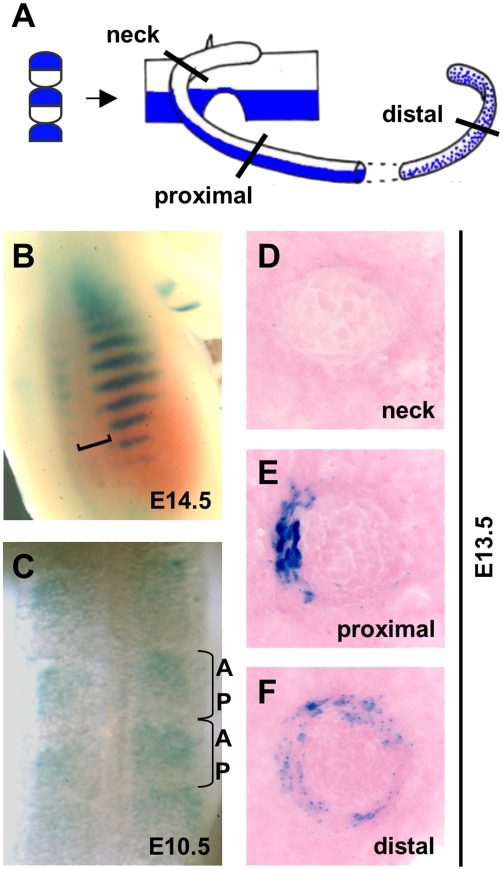
The Ex4r construct drives expression in rib regions that come from anterior somite halves. A. Schematic of chick-quail lineage tracing experiments adapted from [32]. Anterior and posterior somite halves both contribute to developing ribs, with posterior portions (white) giving rise to most of the head and neck region, and anterior somites (blue) contributing specifically to the lateral region of the proximal rib shaft, and more diffusely to the distal rib shaft. B. Dorsal view of an E14.5 Ex4r-*lacZ* transgenic embryo shows a gap (bracket) between the vertebral column and the beginning of rib expression, corresponding to the head and neck region. C. Dorsal view of an E10.5 Ex4r-*lacZ* transgenic embryo shows expression in the anterior region of somites. D–F. Coronal rib cryosections from an E13.5 Ex4r-*lacZ* embryo taken through the neck (D), proximal (E) and distal (F) ribs. Note the lack of expression in the rib neck, the restricted, lateral perichondrial expression in the proximal rib, and the general expression in the distal rib perichondrium.

Ribs are derived from somitic mesoderm [31]. Previous chick-quail lineage tracing experiments have shown that rib cells arise from different portions of developing somites: the head, neck, and the inner surface of ribs are derived from the posterior compartment of somites (white regions in Figure 4A), while the lateral surface of the mid shaft of the rib arises from the anterior compartment of somites (blue regions of Figure 4A) [32]. We noticed that *lacZ* expression driven by the Ex4r construct begins some distance from the vertebral column, and is strongest along the midshaft of ribs (Figure 4B), a pattern reminiscent of the anatomical domain thought to arise from anterior somites.

To analyze rib enhancer activity at additional developmental stages, we generated stable transgenic lines for the Ex4r-*lacZ* construct and collected embryos beginning at embryonic day 10.5. At this early stage of development, the Ex4r-*lacZ* construct is expressed in the anterior halves of developing somites (Figure 4C). Examination of *lacZ* localization in rib sections at later stages showed that Ex4r-driven expression was largely missing from the head and neck region of ribs, was present in the lateral perichondrium along the rib shaft, and became symmetric around the rib in sternal portions (Figure 4D–F). Both the somitic expression and the changes in patterns along the length of ribs suggest that the lateral rib expression reflects the dual origin of ribs from separate somite compartments.

### Altering BMP Signaling in Rib Domains Affects Rib Morphology

Multiple BMP family members are expressed in overlapping patterns in the developing ribs [12]. To test the biological effects of localized increase or decrease in BMP signaling in subdomains of the rib perichondrium, we used the Ex4r sequence to drive the expression of either a constitutively active (*caBmprIb*) or dominant negative (*dnBmprIb*) version of BMP receptor IB [33]. We chose *BmprIb* because it is widely expressed throughout the developing skeleton, including rib perichondrium, and is known to be used by multiple BMP ligands [33]–[38]. Each receptor construct was coinjected with the original Ex4r-*lacZ* clone to generate transgenic embryos.

**Figure 5:**
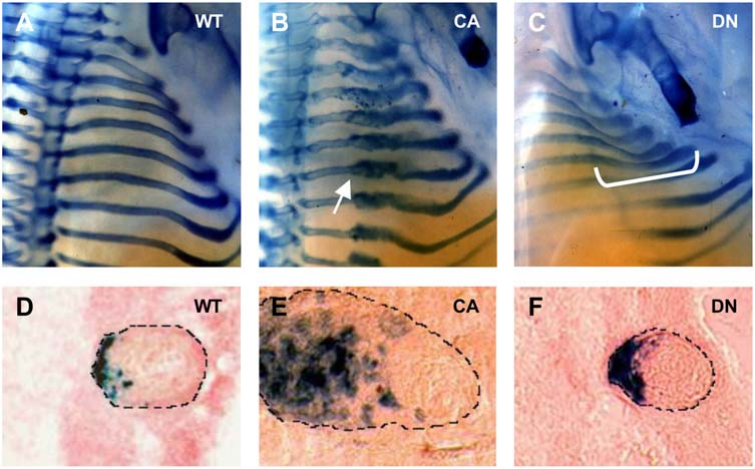
Local changes in BMP signaling alter rib morphology. A–C. Alcian-blue stained rib cages from E14.5 control animals (A), and embryos expressing either a constitutively active (Ex4r-*caBmprIb*) (B) or dominant negative (Ex4r-*dnBmprIb*) (C) form of *BmprIb* under the control of the Ex4r sequence. Increased BMP signaling in the lateral rib perichondrium triggers local rib overgrowth (arrow, B). Decreased BMP signaling alters dorsoventral rib trajectory (bracket, C). D–F. Coronal rib cryosections from Ex4r-*caBmprIb* and Ex4r-*dnBmprIb* transgenic embryos also expressing Ex4r-*lacZ*. Dashed lines denote rib boundaries. D. Normal rib morphology in control embryo. E. The lateral perichondrium forms excess cartilage in Ex4r-*caBmprIb* embryos. F. Decreased BMP signaling in Ex4r-*dnBmprIb* embryos does not affect rib cross-sectional shape.

Both Ex4r-*caBmprIb* and Ex4r-*dnBmprIb* transgenic embryos showed gross changes in rib development at E14.5 when examined by whole-mount skeleton preparations (Figure 5A–C). Increased BMP signaling in the lateral rib domain caused an overgrowth of alcian blue-positive cartilage, beginning midway along the rib shaft (Figure 5B arrow). Sections through Ex4r-*caBmprIb* embryos that were assayed for β-galactosidase activity showed that rib expansion was accompanied by an excess of *lacZ*-positive cells in the lateral rib (Figure 5E). This lateral expression marked the outer edge of the rib deformation (Figure S4A, C) and overlays a cartilaginous mass of cells made up predominantly of hypertrophic chondrocytes expressing *Col2a1* and *Col10a1* (Figure S4E, G) [28],[29]. In contrast, decreased BMP signaling caused a marked deflection in rib trajectory (Figure 5C bracket). Ribs in Ex4r-*dnBmprIb* transgenic embryos emerged normally from the vertebral column but were deflected inwards along the central region of the rib shaft, producing a more constricted upper thorax. This deformation in trajectory was not accompanied by changes in rib cross section (Figure 5F, Figure S4). Neither construct affected the head or neck of the ribs (Figure 5B, C), as expected from the restricted expression domain of Ex4r control sequences along the rib shaft (Figure 4B).

### *Bmp5* Regulatory Mutants Show Domain-Specific Alterations in Rib Growth

**Figure 6:**
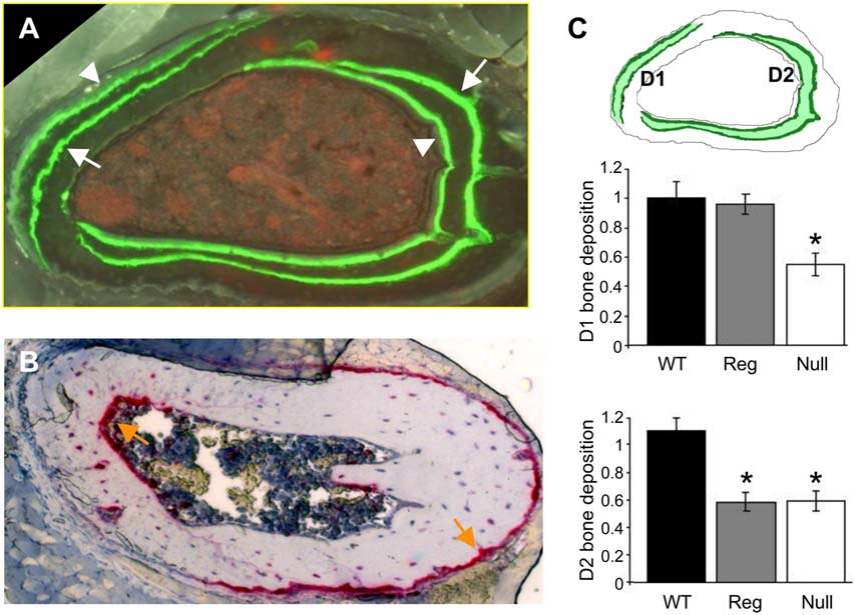
Growth patterns in ribs are highly asymmetric, and are influenced by separate *Bmp5* regulatory regions. A. Bone deposition patterns in growing rib cages of control mice. Calcein dye (green) injected at 6 weeks (arrows) and 7 weeks (arrowheads) produces lines of labeled bone forming at the lateral periosteum (Domain 1 (D1), left) and the anterior, medial, and posterior endosteum (Domain 2 (D2), right) of developing ribs. B. Bone resorption patterns visualized by osteoclast activity. Tartrate-resistant acid phosphatase (red) stains complementary sites of bone erosion (orange arrows) on the lateral endosteum (left) and the anterior, medial, and posterior periosteum (right). C. Schematic of a coronal rib section depicting the area measured (green shade) between 6- and 7-week calcein dye labels to assess relative rates of bone deposition in wild-type, *Bmp5^null^* and *Bmp5^4CHLd^* regulatory mutants. Graphs of measured D1 and D2 areas show impaired bone deposition in both bone deposition domains in *Bmp5^null^* mutants. Only D2 is affected in *Bmp5^4CHLd^* regulatory mutants that lack anterior, medial, and posterior rib elements. Y-axis reflects measured area compare to wild-type, and black asterisks mark significant difference (p<0.05, t-test) from wild-type.

The highly localized domains of *Bmp5* expression in rib perichondrium are reminiscent of previous models suggesting that rib growth occurs by differential activity on the lateral and inner surfaces of the rib [3]. To visualize *in vivo* patterns of bone deposition in growing ribs, we injected mice twice, at 6 and 7 weeks, with calcein, a fluorescent dye that specifically incorporates into newly formed bone (Figure 6). Dorsal rib cross-sections showed two major growth domains labeled with dye; one visible along the lateral periosteal surface (D1), and a second predominantly along the anterior, medial, and posterior endosteal surfaces of the rib (D2). Each bone deposition front is represented by two calcein labelings, reflecting the two separate injections (Figure 6A). Injections with different dye colors demonstrate that the bone fronts labeled by the initial injection (arrows in Figure 6A) become embedded in bone after a week of growth, and that the new bone fronts labeled by the second injection are found near surfaces (arrowheads in Figure 6A, data not shown). These deposition patterns show striking asymmetry, with bone deposition occurring preferentially in the lateral domain of the outer surface periosteum and in the anterior, medial, and posterior domains of the inner endosteum.

To compare patterns of bone deposition and bone resorption, dorsal rib cross-sections were also examined for tartrate-resistant acid phosphatase activity, an osteoclast marker [39]. Bone resorption was also highly asymmetric, and complementary to the areas of bone deposition (Figure 6B). In the outer periosteum, osteoclast activity was most intense on the anterior, medial, and posterior surfaces of the rib; and was nearly absent along the lateral surface where major bone deposition was occurring. Likewise, along the inner endosteum, osteoclast activity was most intense on the lateral wall, and nearly absent from the anterior, medial and posterior surfaces. During growth, these highly asymmetric patterns of bone deposition and resorption would result in the net lateral displacement of ribs and the expansion of the intrathoracic cavity, while preserving marrow space.

*Bmp5* mutant mice are known to have a smaller thoracic volume than wild-type animals [21]. To further characterize detailed bone deposition patterns in *Bmp5* mutants, we performed dual calcein injections on *Bmp5* null and *Bmp5* regulatory mutants, and measured the amount of bone deposition in the different rib domains described above (Figure 6C). Mice with null mutations in the *Bmp5* gene show a significant reduction in bone deposition in both major ossification domains, D1 and D2 (Figure 6C). In contrast, regulatory mutant mice missing anterior, medial and posterior but not lateral rib control sequences (*Bmp5^se4CHLd^*, Figure 1A) show significantly reduced bone deposition in D2, but not in D1 domains. The *Bmp5* gene is thus required for normal rates of bone growth on both the outer and inner surface of the rib, and these two growth domains are controlled independently by different regulatory regions of the *Bmp5* locus.

## Discussion

**Figure 7:**
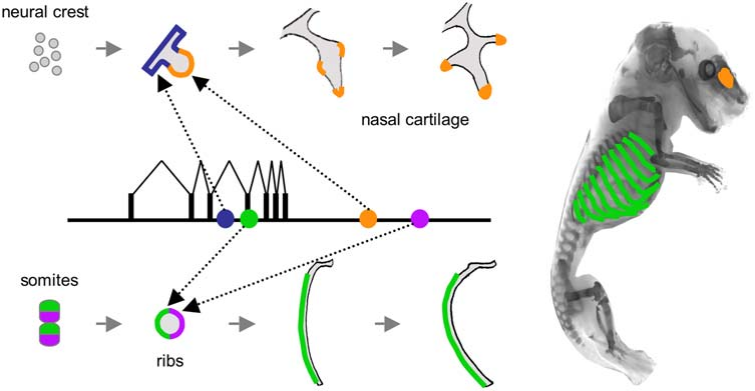
Discrete enhancers control growth in distinct anatomical domains of developing bones. Multiple anatomy-specific enhancers (filled circles) are spread across the *Bmp5* locus. In ribs, two enhancers (green and purple circles) may respond to lineage domains established in somites to control growth on opposing sides of the ribs. Local growth on the lateral edge of rib surfaces promotes rib curvature and expansion of the thoracic cavity. Nasal cartilages form from cranial neural crest. Two enhancers (blue and orange circles) in the *Bmp5* gene are expressed in different highly restricted locations, leading to characteristic branching patterns of the nasal turbinates.

It has long been recognized that cartilage and bone can be molded into a remarkable range of different shapes and sizes. Previous genetic studies show that the morphology of different skeletal elements is controlled by multiple independent genetic factors [2],[40],[41]. Based on studies of jaw and limb morphology in mice, Bailey previously suggested that different subregions of a single bone must be controlled by a large number of independent “morphogenes”, each active in small patches along the surface of a single bone [2]. Despite recent progress identifying genes that regulate formation of all cartilage or all bones, or genes that control skeletal formation in different subdomains along the body axis, little is known about the fine-grained mechanisms that control detailed growth patterns of individual skeletal elements [42]. Here we show that highly defined growth domains in particular bones are controlled by remarkably specific enhancers in the *Bmp5* gene (Figure 7). We propose that anatomy-specific enhancers in BMP genes provide a genomic mechanism for independent developmental control of local growth along discrete domains of individual cartilages and bones in the vertebrate skeleton.

When BMP genes were first discovered and assayed for expression in vertebrates, individual members of the family were initially proposed to promote general steps in the differentiation of all skeletal tissue [8]. Although *Bmp5* is expressed in a continuous fashion in the perichondrial layer surrounding many developing skeletal structures [11],[12], our enhancer surveys do not show evidence for general enhancers in the *Bmp5* gene that drive expression around the surface of all cartilage or all bones. Instead, distinct *Bmp5* enhancers regulate expression in individual skeletal structures. Furthermore, separate enhancers also exist for discrete domains around the surface of individual bones, including lateral, anterior, medial, and posterior domains of the rib perichondrium, and tip versus neck and inter-turbinate domains in the nasal cartilages (Figures 1, 3). This remarkably fine control of gene expression is clearly sufficient to alter skeletal morphology at specific locations (Figure 5). Null and regulatory mutations also show that the *Bmp5* gene is necessary for normal bone deposition rates along particular surfaces of growing ribs (Figure 6). These results confirm that detailed growth patterns in an individual bone can be encoded by highly specific anatomy enhancers in genes for bone morphogenetic proteins.

Previous studies of HOX genes have shown that expression and function at particular anatomical locations in the body are related to the physical location of genes along the chromosome [43]–[45]. The overall correlation between anatomy and gene position may arise from progressive changes in chromatin structure during body axis development; or from proximity to enhancers that map outside the HOX complex, which have decreasing effects on genes that map at increasing physical distances from the enhancer [45],[46]. In contrast, the *Bmp5* skeletal enhancers we have identified to date show no obvious relationship between anatomical position in the body and physical location within the *Bmp5* locus. The regulatory elements for discrete surface domains around a single bone clearly map to different regions of the *Bmp5* gene (Figure 1). In addition, rib and nose enhancers are interspersed with each other (Figure 7) and with other separate enhancer regions previously identified controlling expression in the sternum, thyroid cartilage, lung, and genital tubercle [23],[24].

The dispersed enhancer pattern seen in *Bmp5* may reflect the different roles of BMP and HOX genes in skeletal patterning. Nested sets of HOX gene expression are evolutionarily ancient programs used to pattern basic body axes in both vertebrates and invertebrates [44],[45],[47]. In contrast, both cartilage and bone are more evolutionarily recent, vertebrate-specific tissues that vary widely in form from species to species [1]. For example, respiratory nasal turbinates are thought to have arisen separately in bird and mammals to help conserve water during breathing [48]–[50]. They vary widely in branching structure within mammals, and are reduced or absent in fish, amphibians, and reptiles [48],[51]. Since a variety of studies suggest that BMPs are the endogenous signals used to induce cartilage and bone in vertebrates [7], formation of nasal turbinates and other species-specific skeletal structures presumably occurs through cis- or trans-acting alterations that produce local changes in BMP expression at particular sites in the body. Therefore, the complex architecture of skeletal enhancers in the *Bmp5* gene may reflect a historical process of piecemeal gain and loss of regulatory elements controlling local domains of BMP expression.

How are the remarkably specific domains of *Bmp5* expression generated along the surface of ribs or nasal cartilages? A variety of data suggests that mechanical forces can give rise to highly localized patterns of bone deposition and erosion [52],[53]. For example, rib cages and skulls both enclose rapidly growing tissues. Outward pressure from soft tissue growth may lead to bone deposition on skeletal surfaces under mechanical tension (the convex outermost surface of ribs or cranial bones), and bone erosion on surfaces under compression (the innermost surface of ribs or cranial bones). Although mechanical tension and compression are clearly coupled to bone remodeling, we do not think that the restricted patterns of expression we observe for *Bmp5* enhancers are simply responding to the distribution of mechanical forces on growing skeletal structures. First, there is no obvious relationship between mechanical forces and the contrasting tip and neck expression patterns seen in nasal cartilages. Second, the *Bmp5* enhancer that drives expression along the outer surface of ribs is not similarly expressed along the outer surface of either the sternum or the skull, although these bones should be subject to similar mechanical forces from the rapid expansion of underlying tissue. Third, the Ex4r-*lacZ* construct that drives highly localized patterns of expression in growing ribs also drives compartmentalized expression in developing somites (Figure 4). These results suggest that the remarkably specific *Bmp5* domains in ribs are related to the dual origin of ribs from different somite compartments, rather than to simple mechanical forces acting during later growth and expansion of the thoracic cavity.

Previous lineage tracing experiments have shown that the lateral edges of rib shafts are derived from cells in the anterior half of somites [32] (Figure 4A). Response elements for anterior somite transcription factors could provide a simple mechanism for controlling mid-shaft *Bmp5* expression in the lateral perichondrial domain. Conversely, response elements for posterior somite expression could provide another simple mechanism for regulating *Bmp5* expression in rib head and necks, and in the anterior, medial, and posterior perichondrial domains along the rib shaft, similar to the patterns seen with BAC178 (Figure 1). The current sizes of *Bmp5* rib enhancers are still too large to identify particular binding sites for upstream factors. However, future narrowing of the minimal sequences capable of driving rib domain expression may make it possible to link specific somite transcription factors with the different domains of rib expression identified in this study.

The dual origin of axial structures from anterior and posterior halves of adjacent somites produces vertebrae and ribs that form one half segment out of register with the original metameric pattern seen in somites. The functional significance of this shift has been debated for over a hundred years [31], [54]–[56]. Resegmentation causes axial muscles, and many of their origin and insertion points on adjacent vertebrae and ribs, to all be derived from a single somite. Our studies suggest resegmentation also plays a key role in establishing detailed growth patterns in developing ribs (Figure 7). Although ribs are usually thought of as simple tubular structures, they can be extensively modified in different organisms to produce the diverse cross-sectional shapes, as well as the varied curvatures seen in wide- and narrow-bodied animals [1],[57]. It has long been recognized that differential deposition on the lateral surface of ribs must underlie the expansion and ultimate shape of ribs and thoracic cavities [3]. We suggest that resegmentation helps establish the lineage domains that make it possible to independently control cartilage and bone growth in specific rib surface domains. The multiple enhancers present in BMP genes provide an elegant mechanism for linking such lineage domains to actual sites of bone growth, leading to highly detailed patterns of deposition that can be independently controlled along the length and around the circumference of a single bone.

While lineage domains may be used to produce separate lateral versus medial domains of gene expression in developing ribs, we think additional mechanisms must be operating to produce other highly localized patches of *Bmp5* expression. For example, our comparison of BAC199 and BAC178 expression suggests at least four different expression domains may exist at certain positions along the ribs (lateral, medial, anterior, posterior; Figure 1). Control sequences for the lateral domain have been mapped to a single 1069 bp peak of sequence conservation within the *Bmp5* Ex4r region, but additional sequences responsible for expression in the other domains remain to be identified in the larger regions covered by BAC199 and BAC178.

Highly localized expression patterns are also seen in multiple spatially restricted patches along the necks and tips of nasal cartilages (Figure 3). The elements controlling these patches are distinct from those controlling rib expression. In addition, nasal cartilage development is quite different from rib morphogenesis (Figure 7). For example, the facial bones and cartilages are derived from cranial neural crest that migrates from positions in the developing brain [58]–[60]. HOX genes are not expressed in this cranial region, and transplantation studies have demonstrated a remarkable degree of plasticity in the cranial neural crest populations [61],[62]. Patterning signals are thought to emerge from the local endoderm and ectoderm to control the shape and size of individual facial skeletal structures [61],[63]. Therefore, unlike ribs, we currently know of no lineage compartments that can account for the various separate tip and shelf domains seen during the branching morphogenesis of nasal cartilages.

In *Drosophila*, branching morphogenesis takes place during tracheal airway development, and is controlled by numerous local patches of *breathless*/FGF expression. The specific enhancers controlling *breathless* expression near tips of growing trachea branches have not been isolated, but may respond to different combinations of transcription factors that are themselves expressed in local or intersecting patterns [64]. Multiple, locally acting enhancers in BMP and FGF genes may thus represent a common molecular strategy for molding skeletal tissue or trachea airways into particular shapes in different animals [7],[64].

Further studies of anatomy-specific elements in BMP genes should lead to a molecular understanding of the type of “morphogenes” that have long been postulated to control local growth decisions in different subdomains of particular bones [2]. In addition, gain and loss of regulatory elements in BMP genes may provide a simple genomic mechanism for evolutionary modification of skeletal structures. While null mutations in BMP genes often have pleiotropic defects, adaptive changes in specific regulatory sequences could localize effects to particular skeletal structures, making it possible to alter vertebrate anatomy while preserving viability and fitness [7]. This possibility has taken on renewed interest in light of studies linking changes in BMP expression to different beak shapes in naturally occurring bird species [65]–[67], and to different jaw morphologies in African cichlids [68]. Regulatory lesions are difficult to identify, and it has not yet been possible to track particular bird or fish anatomical changes to specific DNA sequence alterations in BMP genes. Nonetheless we think the kind of modular regulatory architecture we have found for the *Bmp5* gene probably exists around many other members of the BMP family [69],[70]. Isolation and characterization of additional anatomy elements from BMP genes will make it possible to test whether anatomical changes in naturally occurring species result from structural and functional modifications in the type of modular enhancer regions identified in this study.

## Materials and Methods

### Mouse Strains and Transgenics

Regulatory (*Bmp5^se38DSD^*, *Bmp5^se4CHLd^)* and null (*Bmp5^null^)* alleles were described previously [11],[22],[23]. All strains used for bone growth assays are on the C57Bl/6J background. The generation of BAC199-*lacZ*, BAC178-*lacZ* and Phage7-*lacZ* transgenics was reported in [23],[24]. All new DNA constructs were prepared for microinjection as previously described [24]. The Ex4r-*caBmprIb* and Ex4r-*dnBmprIb* plasmids were coinjected with the Ex4r-*lacZ* clone at a 4∶1 molar ratio. Pronuclear injection into FVB embryos was carried out by the Stanford Transgenic Facility and Xenogen Biosciences in accordance with protocols approved by the Stanford University Institutional Animal Care and Use Committee.

### *Bmp5* Sequencing and Comparative Analysis

BAC426K2 (Genbank accession #AC079245) and BAC343K17 (Genbank Accession #AC079244) were isolated from the RPCI-23 Female (C57Bl/6J) Mouse BAC library (Invitrogen) using a 1334 bp *EcoR*I probe and/or a 591 bp *Hae*III probe located 123,536 bp and 225,112 bp, respectively, from the *Bmp5* transcriptional start site. Sequences were compiled following designation of BAC426K2 and BAC343K17 as clones of high biomedical interest by the National Human Genome Research Institute and sequencing by the Advanced Center for Genome Technology at the University of Oklahoma, Norman. 5′ mouse sequences were added from BAC429A10 (Genbank accession #AC144940) as they became available. Human *Bmp5* genomic sequence was compiled from the following clones: Genbank accession numbers AL589796, AL137178, AL133386, AL590290, AL590406 and AL592426. The human and mouse *Bmp5* sequences were masked using RepeatMasker (A.F.A. Smit, R. Hubley and P. Green, unpubl.; http://www.repeatmasker.org/). ECRs were identified using global sequence alignment programs as previously described [69].

### Plasmid Construction

The Ex4r-*lacZ* plasmid was generated by amplifying a 6221 bp fragment corresponding to mouse sequences 93,656–99,876 bp in Figure 2 using primers 622: 5′GGATTGCGGCCGCTATGGACAGCTTTGAAGAGCTTTGGTA3′ and 624: 5′GGATTGCGGCCGCTATTCTAGCCTCTCCTGTAGGATTATG3′. Following *Not*I digestion, the fragment was cloned into the *Not*5'hsp*lacZ* vector [23].

To generate Ex4r-*caBmprIb* and Ex4r-*dnBmprIb* constructs, the constitutively active (ca) or dominant negative (dn) form of *BmprIb* was amplified using primers lpf21: 5′CATGCCATGGCCATGCTCTTACGAAGCTCTGGAAAAT3′ and lpf22: 5′GCTCTAGAGCTTAGATCCCCCCTGCCCGGTTATTATTATCAGAGTTTAATGTCCTGGGACTCTG3′. The PCR products were digested with *Nco*I/*Xba*I and cloned into the Ex4r-*lacZ* plasmid that had been digested with *Nco*I and *Xba*I (partial), replacing the *lacZ* cassette.

To generate plasmid Ex4rCD-*lacZ*, a 3 kb fragment was amplified from the *Bmp5* BAC426K2 using primers 624 (above) and 627: 5′GGATTGCGGCCGCTATTCTAGGCTGTTGGAAAGCAAGTCTA3′. The PCR product was digested with *Not*I and cloned into *Not*5'hsp*lacZ*.

Construct Ex4rΔC-*lacZ* was generated using primers 750: 5′ATGTGGCCAAACAGGCCTATTAATGGTCAACCAGATGAATACAGCA3′ and 751: 5′ATGTGGCCTGTTTGGCCTATTATAGAACACATAGAGGCATACCAGG3′ to amplify directly from the Ex4r-*lacZ* plasmid using the Expand Long Template PCR system (Roche #1681834). The 12.9 kb product was digested with *Sfi*I and the free ends were ligated together. The 5488 bp insert with a 733 bp deletion of ECR C from Ex4r was removed by *Not*I digestion and recloned into an unamplified *Not*5'hsp*lacZ* vector.

The Ex4rΔD-*lacZ* plasmid was generated by amplifying 4677 kb and 454 bp products from BAC426K2 using primer 622 (above) with primer 754: 5′ATGTGGCCTGTTTGGCCTATTCCTTTTGAGAATCTCGGCTTCTAGA3′ and primer 752: 5′ATGTGGCCAAACAGGCCTATTGGCAGGTTAGAGAAAGTAATGATAG3′ with primer 624 (above), respectively. The PCR products were digested with *Sfi*I and ligated together. The resulting 5.1 kb product containing a 1069 bp deletion of ECR D from Ex4r was digested with *Not*I and ligated into the *Not*5'hsp*lacZ* vector.

Plasmid ECRD-*lacZ* was generated by amplifying a 1127 bp fragment from BAC426K2 using primers 690: 5′ATGTGGCCTGTTTGGCCTATTCTTTCTCTAACCTGCCTCTACCCTG3′ and 736: 5′ATGTGGCCAAACAGGCCTATTGAAGCCGAGATTCTCAAAAGGTGGA3′. The PCR product was digested with *Sfi*I and ligated into p*Sfi*-hsp*lacZ* [69].

### *lacZ* Detection

Embryos collected by Xenogen Biosciences were fixed for 1 hour in 4% paraformaldehyde in 1× PBS at 4°C, placed in cold 1× PBS and shipped overnight on ice.

Whole-mount staining for β-galactosidase activity was performed as described [69] with the following modifications: Embryo fixation times varied with age (E10.5 for 30 minutes, E13.5 for 75 minutes, E14.5 for 90 minutes). E13.5-E14.5 embryos were hemisected after 1 hour. Rib and nasal cartilage cryosections from *lacZ* whole-mount embryos were collected and counterstained as described [69]. Prior to embedding, samples were equilibrated in embedding solution (15% sucrose, 7.5% gelatin (300 Bloom, Sigma #G2500) in 1× PBS) for 1 hour at 42°C. Ex4r-*caBmprIb* and Ex4r-*dnBmprIb* transgenic embryos were frozen in OCT compound (Tissue Tek), cryosectioned at 25 microns and counterstained with Nuclear Fast Red (Vector labs, #H-3403).

β-galactosidase activity on cryosections was assayed by fixing samples in 4% paraformaldehyde in 1× PBS for 5–8 minutes at room temperature. Slides were rinsed 3 X 5 minutes with 1× PBS, washed in *lacZ* wash buffer (0.1 M sodium phosphate buffer (pH 7.3), 2 mM MgCl, 0.01% deoxycholate, 0.02% Nonidet P-40) for 10 minutes and incubated in *lacZ* stain (wash buffer supplemented with 4 mM K_3_Fe(CN)_6_, 4 mM K_4_Fe(CN)_6_⋅3 H_2_O, 0.1M Tris (pH 7.4) and 1 mg/mL X-gal (Sigma #B4252)) at 37°C for at least 24 hours. Stained sections were rinsed with 1× PBS, fixed for an additional 10 minutes in 4% paraformaldehyde in 1× PBS, and counterstained with Nuclear Fast Red.

### *In Situ* Hybridization

The *Bmp5, Col10a1,* and *Col2a1* probes used were described [23],[29],[71]. The *MyoD1* probe was generated from a clone ordered from Open Biosystems (clone id 372340). The *Col1a1* probe was generated using the pM*ColI*-*Bam* plasmid (a gift of Dr. Ernst Reichenberger). Timed matings were performed to collect wild-type (C57Bl/6J), Ex4r-*lacZ*, Phage7-*lacZ*, and *Bmp5^se38DSD^* mutant heads and/or torsos at E13.5-E15.5. Ex4r-*caBmprIb* and Ex4r-*dnBmprIb* embryos generated by Xenogen Biosciences were collected at E15.5 and fixed for 1 hour in 4% paraformaldehyde in 1× PBS, bisected, and one half embryo embedded in OCT and one half analyzed for β-galactosidase activity to identify transgenic embryos. 12 micron sections were collected from samples frozen in OCT compound and analyzed for gene expression as previously described [72]; except that the color reagent BM purple (Roche #1442074) was used in place of NBT/BCIP.

### Embryonic Skeletal Analysis

E14.5 skeletons were prepared as described [73], with the following modifications: Embryos were placed directly into staining solution after ethanol dehydration. Following potassium hydroxide treatment, embryos were cleared in 50% glycerol overnight, and then stored in 100% glycerol. All steps were done at room temperature.

### Rib Growth Analysis

Two successive intraperitoneal injections of calcein (Sigma # C0875, 2.5 mg/ml in 1× PBS) were performed at postnatal day 43 (p43) and p51 on C57Bl/6J males (10 mg injected/kg body weight). Whole rib cages were collected at p53 and dehydrated in ethanol for at least 1 week at 4°C, then embedded in methylmethacrylate and ground sectioned to obtain 50 micron coronal sections by HMAC (Birmingham, AL). To quantify levels of bone deposition in wild-type and mutant animals, calcein labeled rib cages from six males of each category (C57Bl/6J, *Bmp5^se4CHLd^* and *Bmp5^null^*) were equilibrated overnight in 15% sucrose in 1× PBS and at least 24 hours in 30% sucrose in 1× PBS, all at 4°C. Rib cages were bisected, and the right half was embedded in OCT. Six 50 micron coronal cryosections were taken approximately 1 mm apart, beginning at the growth plate and moving dorsally. Each section was digitally photographed, and pixel areas between labeled bone deposition fronts were measured with Photoshop. All measurements were taken on the fifth rib. Data are expressed as mean areas±s.e.m. relative to wild-type mice. Differences between groups were evaluated using Student's *t*-test.

### Tartrate-Resistant Acid Phosphatase Stains

C57Bl/6J male rib cages were collected at p53 into cold 1× PBS, fixed in 4% paraformaldehyde in 1× PBS for 3 days at 4°C, and washed 3 times for 30 minutes in cold 1× PBS. The right halves were embedded in paraffin, sectioned, and stained by HMAC [74].

### MicroCT Analysis

Scans from 4 wild-type and 5 *Bmp5^null^* mutant skulls, aged 4 weeks postnatally, were generated using a Scanco MicroCT-40 operated at a tube potential of 45 kV and tube current of 177 microA using a 0.30 second integration with 2× averaging. All samples had undergone skeletal preparation prior to scanning.

## Supporting Information

Figure S1 Enhancer survey of the 3′ *Bmp5* regulatory region. Global sequence comparison of the mouse and human *Bmp5* loci by LAGAN/VISTA beginning approximately 150 kb downstream of the transcriptional start site of *Bmp5*. Blue peaks denote exons of *Hmgcll1*, the gene immediately downstream of *Bmp5*. ECRs of 70% identity over at least 300 bp are highlighted (pink). Percent sequence identity between mouse and human sequence (Y-axis) ranges from 50–100%. The gray brackets show ECR constructs tested in transgenic assays.
(1.54 MB TIF)

Figure S2 Narrowing of the lateral rib enhancer. A VISTA plot derived from a mouse/human sequence comparison shows four conserved sequences (ECRs A, B, C and D) within the 6.2 kb Ex4r subclone. Coordinates reflect the position in the mouse *Bmp5* locus. Below are schematics of five constructs used for transgenic analysis to narrow the region of the lateral rib enhancer. Constructs tested were 1) Ex4r-*lacZ*; 2) Ex4rCD-*lacZ*, a 3 kb subclone including Exon 4 and ECRs C and D; 3) Ex4rΔC-*lacZ*, a 733 bp deletion of Exon 4 and ECR C from the Ex4r subclone; 4) Ex4rΔD-*lacZ*, a 1069 bp deletion of ECR D from the Ex4r subclone; 5) ECRD-*lacZ*, a 1127 bp subclone of ECR D. **1–5**. β-galactosidase activity in representative embryos generated from constructs 1–5 at E13.5 or E14.5. Insets show a coronal rib section from the embryo pictured. 2, 3. ECRs A–C are not required for lateral rib expression. 4, 5. Sequences corresponding to ECR D are both necessary (4) and sufficient (5) to control lateral rib expression. Note, removal of ECR D does not affect limb or nasal cartilage expression (arrows and asterisk in 4).
(6.07 MB TIF)

Figure S3 *Bmp5* nasal regulatory domains colocalize with cartilage markers. A–F. Coronal cryosections through the developing nasal cartilages analyzed for β-galactosidase activity (A, B), *Col1a1* (C, D) or *Col2a1* (E, F) expression. A, C, E. Near adjacent sections from an Ex4r-*lacZ* embryo at E15.5. Comparison of panels A and C shows the turbinate neck expression controlled by the Ex4r subclone overlaps the domain of *Col1a1* expression along the surface of the developing turbinate (arrows, C). B, D, F. Near adjacent sections from a Phage7-*lacZ* embryo at E15.5. Comparison of panels B and F shows the expression driven by Phage 7 sequences overlaps *Col2a1* expressing chondrocytes at the tip of the growing turbinate.
(3.42 MB TIF)

Figure S4 Ex4r-*caBmprIb* causes laterally-directed cartilage rib extensions. A–H. Coronal rib cryosections analyzed for β-galactosidase activity (A, B) or *Col1a1* (C, D), *Col10a1* (E, F), or *Col2a1* (G, H) expression. A, C, E, G. Near adjacent rib sections from an Ex4r-*caBmprIb* embryo at E15.5. The dashed lines correspond to an enlarged mass extending from the lateral quadrant of the developing rib. β-galactosidase activity (A) is found predominantly at the lateral edge of the expanded mass, in perichondrial cells also expressing *Col1a1* (C). E, G. The mass itself is composed of developing chondrocytes expressing *Col10a1* (E) and *Col2a1* (G). B, D, F, H. Near adjacent rib sections from an Ex4r-*dnBmprIb* embryo at E15.5. β-galactosidase activity (B) is restricted to the perichondrial cells expressing *Col1a1* (D). No differences from wild type rib developmental expression patterns were observed.
(6.50 MB TIF)
