# Supplementary figures and images for: Shaping Skeletal Growth by Modular Regulatory Elements in the Bmp5 Gene
Source: PLoS Genet. 2008 Dec 19;4(12):e1000308. doi: 10.1371/journal.pgen.1000308 (PMC2592695; doi:10.1371/journal.pgen.1000308)

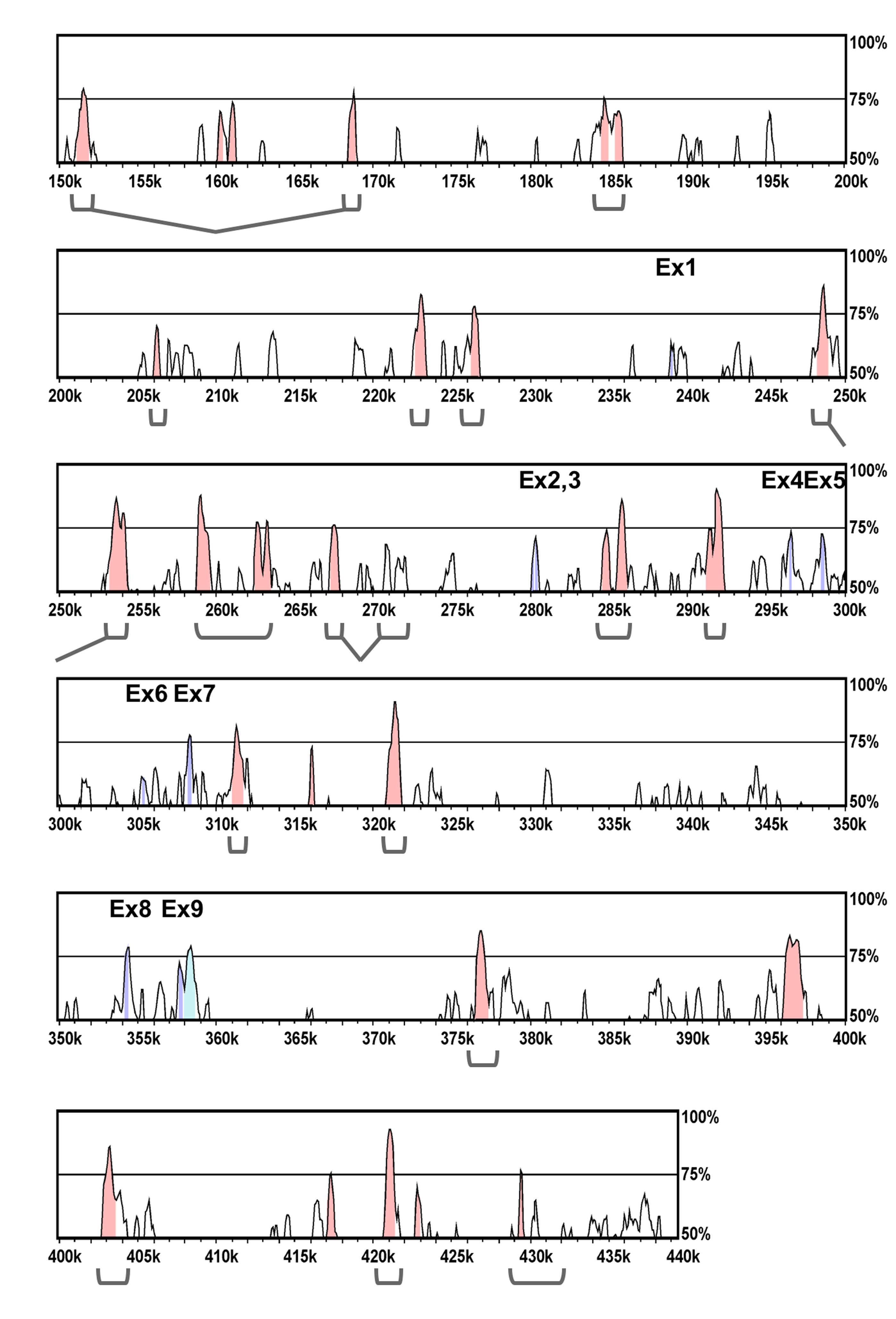

Supplement: Figure S1 — Enhancer survey of the 3′ Bmp5 regulatory region. Global sequence comparison of the mouse and human Bmp5 loci by LAGAN/VISTA beginning approximately 150 kb downstream of the transcriptional start site of Bmp5. Blue peaks denote exons of Hmgcll1, the gene immediately downstream of Bmp5. ECRs of 70% identity over at least 300 bp are highlighted (pink). Percent sequence identity between mouse and human sequence (Y-axis) ranges from 50–100%. The gray brackets show ECR constructs tested in transgenic assays. (1.54 MB TIF) [file pgen.1000308.s001.tif]

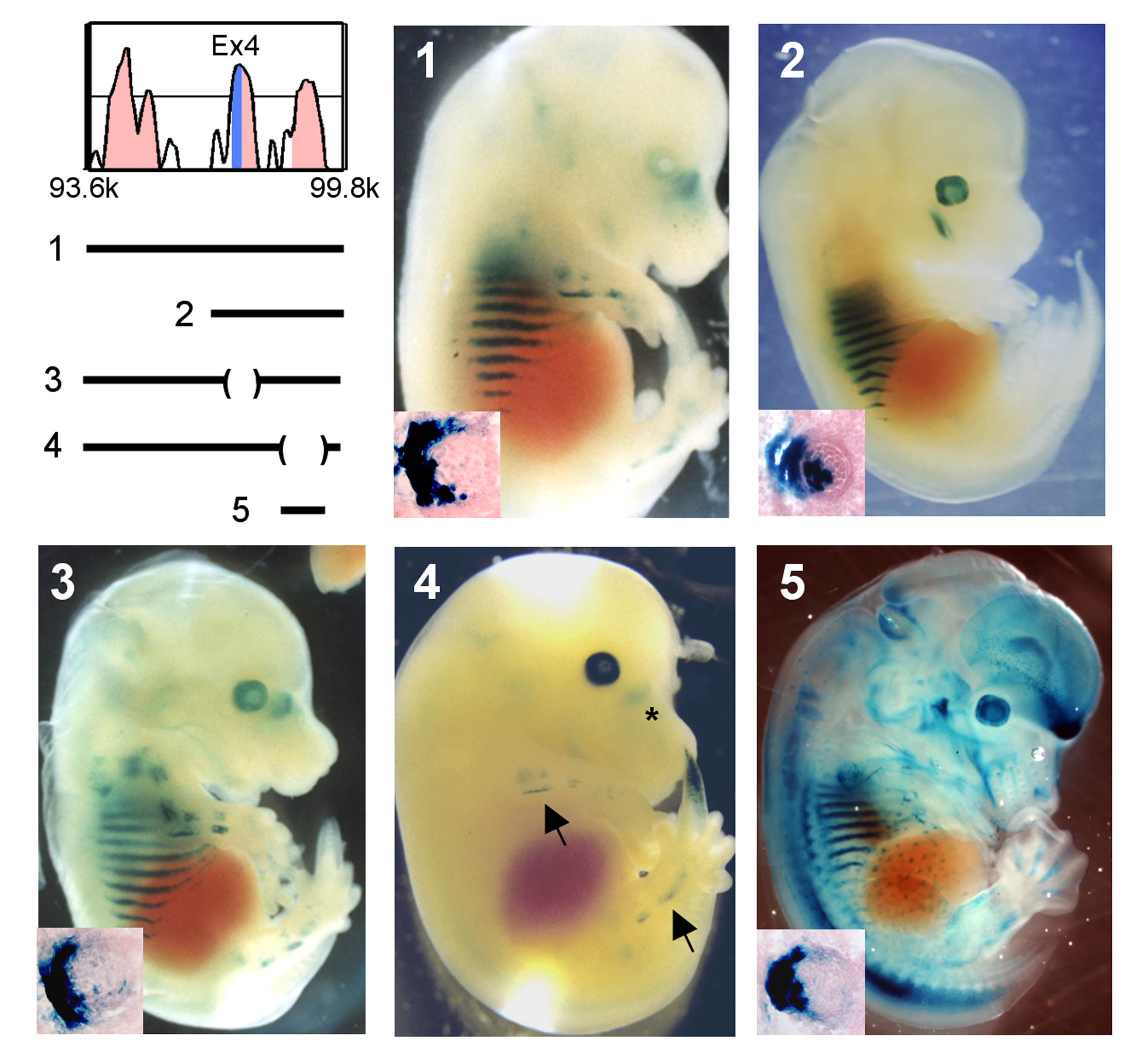

Supplement: Figure S2 — Narrowing of the lateral rib enhancer. A VISTA plot derived from a mouse/human sequence comparison shows four conserved sequences (ECRs A, B, C and D) within the 6.2 kb Ex4r subclone. Coordinates reflect the position in the mouse Bmp5 locus. Below are schematics of five constructs used for transgenic analysis to narrow the region of the lateral rib enhancer. Constructs tested were 1) Ex4r-lacZ; 2) Ex4rCD-lacZ, a 3 kb subclone including Exon 4 and ECRs C and D; 3) Ex4rΔC-lacZ, a 733 bp deletion of Exon 4 and ECR C from the Ex4r subclone; 4) Ex4rΔD-lacZ, a 1069 bp deletion of ECR D from the Ex4r subclone; 5) ECRD-lacZ, a 1127 bp subclone of ECR D. 1–5. β-galactosidase activity in representative embryos generated from constructs 1–5 at E13.5 or E14.5. Insets show a coronal rib section from the embryo pictured. 2, 3. ECRs A–C are not required for lateral rib expression. 4, 5. Sequences corresponding to ECR D are both necessary (4) and sufficient (5) to control lateral rib expression. Note, removal of ECR D does not affect limb or nasal cartilage expression (arrows and asterisk in 4). (6.07 MB TIF) [file pgen.1000308.s002.tif]

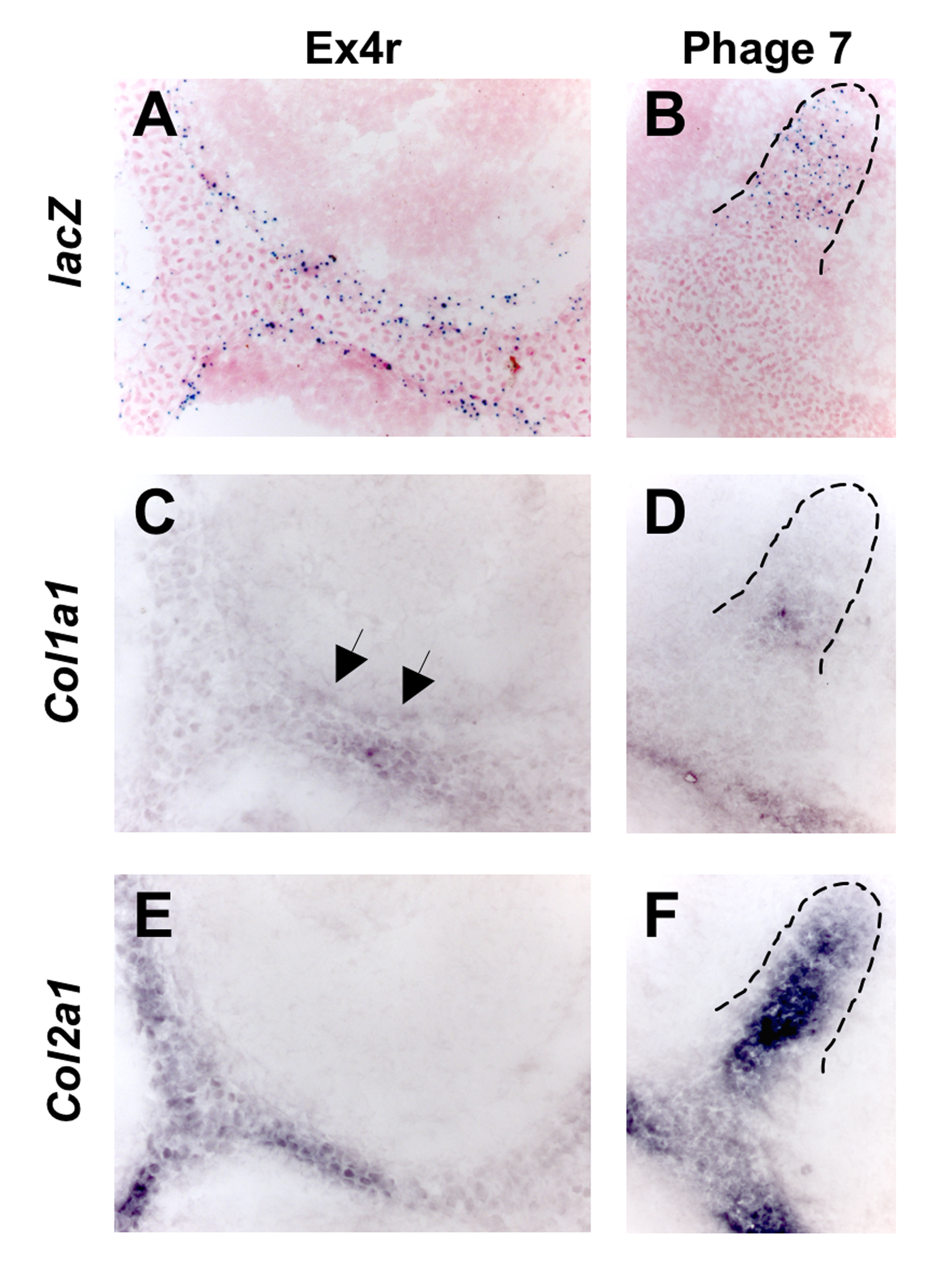

Supplement: Figure S3 — Bmp5 nasal regulatory domains colocalize with cartilage markers. A–F. Coronal cryosections through the developing nasal cartilages analyzed for β-galactosidase activity (A, B), Col1a1 (C, D) or Col2a1 (E, F) expression. A, C, E. Near adjacent sections from an Ex4r-lacZ embryo at E15.5. Comparison of panels A and C shows the turbinate neck expression controlled by the Ex4r subclone overlaps the domain of Col1a1 expression along the surface of the developing turbinate (arrows, C). B, D, F. Near adjacent sections from a Phage7-lacZ embryo at E15.5. Comparison of panels B and F shows the expression driven by Phage 7 sequences overlaps Col2a1 expressing chondrocytes at the tip of the growing turbinate. (3.42 MB TIF) [file pgen.1000308.s003.tif]

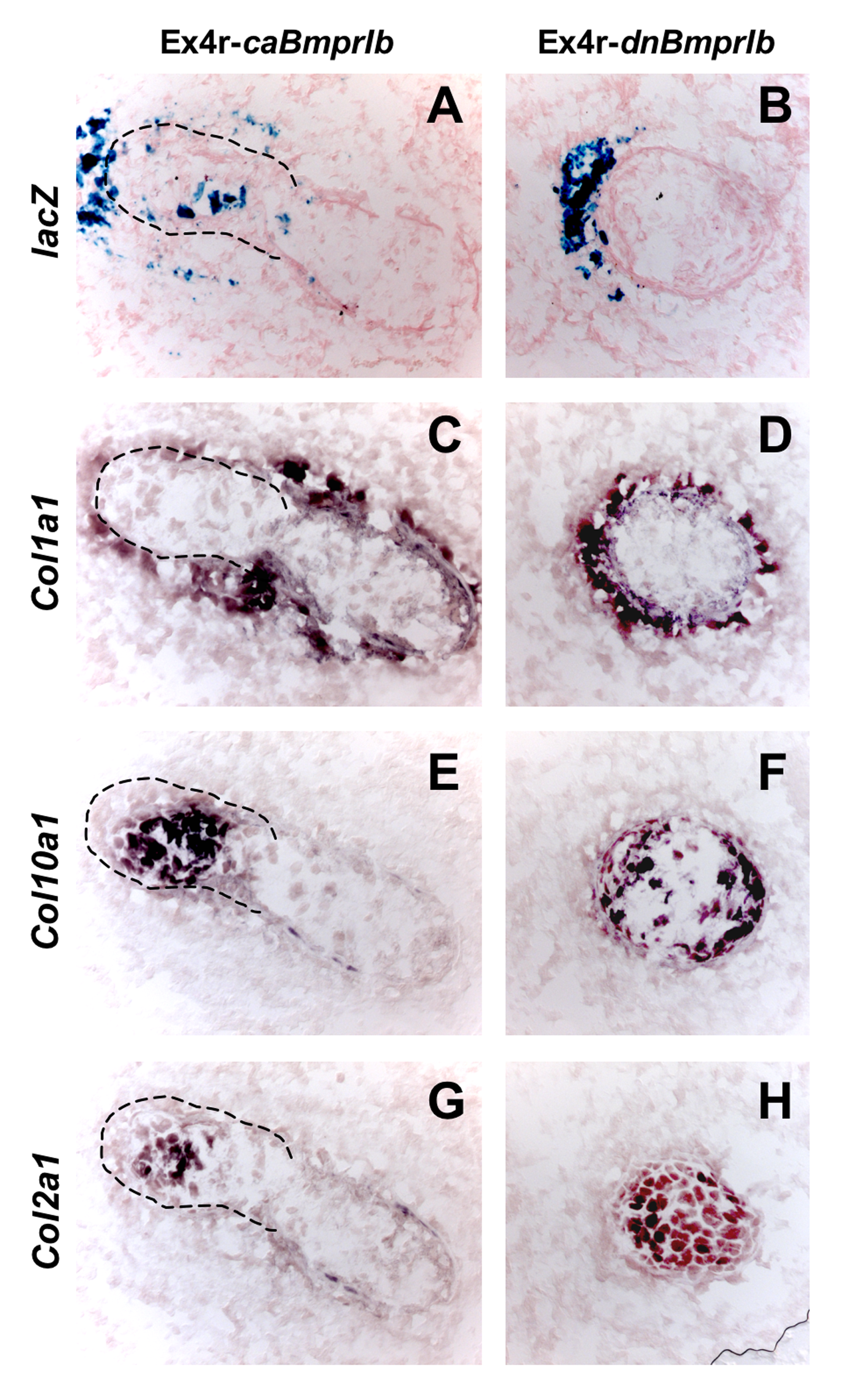

Supplement: Figure S4 — Ex4r-caBmprIb causes laterally-directed cartilage rib extensions. A–H. Coronal rib cryosections analyzed for β-galactosidase activity (A, B) or Col1a1 (C, D), Col10a1 (E, F), or Col2a1 (G, H) expression. A, C, E, G. Near adjacent rib sections from an Ex4r-caBmprIb embryo at E15.5. The dashed lines correspond to an enlarged mass extending from the lateral quadrant of the developing rib. β-galactosidase activity (A) is found predominantly at the lateral edge of the expanded mass, in perichondrial cells also expressing Col1a1 (C). E, G. The mass itself is composed of developing chondrocytes expressing Col10a1 (E) and Col2a1 (G). B, D, F, H. Near adjacent rib sections from an Ex4r-dnBmprIb embryo at E15.5. β-galactosidase activity (B) is restricted to the perichondrial cells expressing Col1a1 (D). No differences from wild type rib developmental expression patterns were observed. (6.50 MB TIF) [file pgen.1000308.s004.tif]
